# Impact of Community-Based Lymphedema Management on Perceived Disability among Patients with Lymphatic Filariasis in Orissa State, India

**DOI:** 10.1371/journal.pntd.0002100

**Published:** 2013-03-14

**Authors:** Philip J. Budge, Kristen M. Little, Katherine E. Mues, Erin D. Kennedy, Aiysha Prakash, Jonathan Rout, LeAnne M. Fox

**Affiliations:** 1 Epidemic Intelligence Service, Centers for Disease Control and Prevention, Atlanta, Georgia, United States of America; 2 Parasitic Diseases Branch, Division of Parasitic Diseases and Malaria, Center for Global Health, Centers for Disease Control and Prevention, Atlanta, Georgia, United States of America; 3 Department of Epidemiology, Rollins School of Public Health, Emory University, Atlanta, Georgia, United States of America; 4 Church's Auxiliary for Social Action, East Zone, Kolkata, India; National Institute of Parasitic Diseases China Center for Disease Control and Prevention, China

## Abstract

**Background:**

Lymphatic filariasis (LF) infects approximately 120 million people worldwide. As many as 40 million have symptoms of LF disease, including lymphedema, elephantiasis, and hydrocele. India constitutes approximately 45% of the world's burden of LF. The Indian NGO Church's Auxiliary for Social Action (CASA) has been conducting a community-based lymphedema management program in Orissa State since 2007 that aims to reduce the morbidity associated with lymphedema and elephantiasis. The objective of this analysis is to evaluate the effects of this program on lymphedema patients' perceived disability.

**Methodology/Principal Findings:**

For this prospective cohort study, 370 patients ≥14 years of age, who reported lymphedema lasting more than three months in one or both legs, were recruited from villages in the Bolagarh sub-district, Khurda District, Orissa, India. The World Health Organization Disability Assessment Schedule II was administered to participants at baseline (July, 2009), and then at regular intervals through 24 months (July, 2011), to assess patients' perceived disability. Disability scores decreased significantly (p<0.0001) from baseline to 24 months. Multivariable analysis using mixed effects modeling found that employment and time in the program were significantly associated with lower disability scores after two years of program involvement. Older age, female gender, the presence of other chronic health conditions, moderate (Stage 3) or advanced (Stage 4–7) lymphedema, reporting an adenolymphangitis (ADL) episode during the previous 30 days, and the presence of inter-digital lesions were associated with higher disability scores. Patients with moderate or advanced lymphedema experienced greater improvements in perceived disability over time. Patients participating in the program for at least 12 months also reported losing 2.5 fewer work days per month (p<0.001) due to their lymphedema, compared to baseline.

**Significance:**

These results indicate that community-based lymphedema management programs can reduce disability and prevent days of work lost. These effects were sustained over a 24 month period.

## Author Summary

Lymphatic filariasis (LF) is the world's second-leading cause of disability and causes limb lymphedema and elephantiasis in up to 15 million people and lymphedema or hydrocele in over 40 million people, worldwide. A massive global effort has been undertaken to eliminate LF as a public health problem. LF elimination is based on two pillars: (1) interruption of transmission and (2) treatment of clinical disease among those already affected. The Indian NGO, Church's Auxiliary for Social Action (CASA), has been providing community-based treatment of lymphedema in Khurda District, Orissa State, India, since 2007. We evaluated the impact of this treatment program on the participating patients' perceived disability using the WHO Disability Assessment Schedule II (WHO-DAS II). After two years of enrollment in the program, patients had significantly lower levels of perceived disability. We found that being employed and time enrolled in the program were associated with significant reductions in disability scores. Compared to baseline, patients enrolled in the program for at least 12 months reported 2.5 fewer days of work lost in the previous 30 days due to their lymphedema. These findings indicate that participation in a community-based lymphedema management program can reduce patients' disability and prevent days of work lost due to lymphedema.

## Introduction

Lymphatic filariasis (LF) is a parasitic infection that leads to damage of the lymphatic system, causing lymphedema of the legs, arms, breast, or genitals. These symptoms affect an estimated 40 million people, making LF the second-leading cause of disability globally [1], [2]. Despite remarkable progress toward the interruption of LF transmission [3], less attention has been paid to LF morbidity management and disability prevention, which remain critical problems in many endemic areas [4].

Though filarial infection causes initial lymphatic dysfunction, development and progression of lymphedema is thought to result from recurrent episodes of secondary bacterial infections, known as adenolymphangitis (ADL). Patients with lymphatic damage are at increased risk for ADL episodes due to poor lymphatic drainage and predisposition to interdigital fungal infections, which can serve as a portal of entry for pathogenic bacteria [5]. ADL episodes are characterized by pain, swelling, and inflammation of the affected extremity, often accompanied by fever or chills. These episodes further damage lymphatic vessels and worsen lymphatic dysfunction, leading to an increased risk for additional ADL episodes [5].

While LF-associated lymphedema cannot be completely cured, low-cost, effective approaches to morbidity management are available for lymphedema patients [6], [7]. Proper care of lymphedema, known as lymphedema management, has been shown to be effective in preventing disease progression, reducing limb swelling, and reducing the frequency of ADL episodes [6]–[8]. Lymphedema management includes regular limb washing, appropriate exercise, elevation of the affected limb, early treatment of bacterial and fungal infections, and use of proper footwear [5].

Morbidity control is of special concern in India, where an estimated 59 million people are infected with the parasites that cause lymphatic filariasis, approximately 19.6 million of whom exhibit symptoms of lymphedema, elephantiasis, or hydrocele [9]. LF predominately affects the poorest segments of India's population, and the associated morbidity and disability are compounded by stigmatization, strict caste and gender roles, and a lack of access to healthcare [10]–[12].

**Figure 1:**
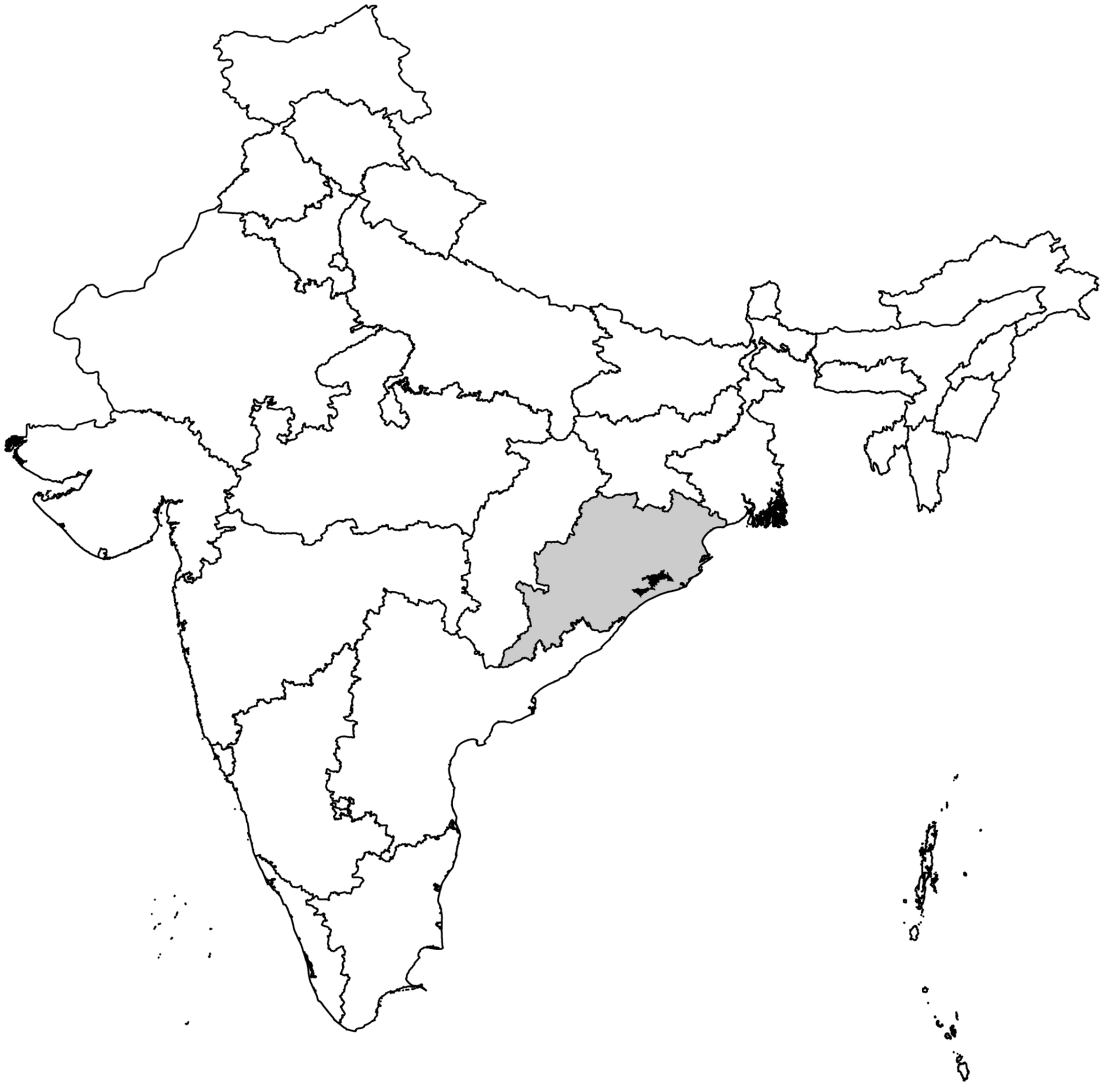
Map of India. The location of Orissa state (shaded gray) and Khurda District (shaded black) are indicated.

Since 2007, the Indian non-governmental organization (NGO) Church's Auxiliary for Social Action (CASA), has been providing community-based treatment of lymphedema in Orissa State, India (**Figure 1**) [13]. The program currently serves more than 20,000 lymphedema patients and their families through a network of village volunteers, who are trained to provide home-based care and instruction in lymphedema management techniques.

While previous studies have demonstrated improvements in patient quality of life and a reduction in ADL episodes after beginning lymphedema management, most have assessed patients over relatively short periods of time (≤1 year) and on a smaller scale [14], [15]. The objective of this study, therefore, was to evaluate the longer-term impact of a large-scale, community-based lymphedema management program on perceived disability and productivity among lymphedema patients using a validated disability-assessment tool [16].

## Methods

### Ethics Statement

This project was submitted for human subjects review to the Center for Global Health at the Centers for Disease Control and Prevention (CDC), Atlanta, Georgia, USA. The project was determined to be program evaluation under CDC policy prior to the implementation of the survey. Permission for the survey was obtained from the Orissa State Department of Health and Family Welfare. Participants were asked to give their written consent prior to participation. For those unable to write, consent was documented by recording the person's fingerprint or marking the signature line with an ‘X’ and by countersignature of survey personnel. For participants under 18 years of age, verbal consent of a parent or guardian was also obtained. Consent procedures were approved by CDC and the Orissa State Department of Health and Family Welfare.

### Study Area

Khurda District, Orissa State, India, is located near India's east coast on the northern portion of the Bay of Bengal (**Figure 1**), and contains the state capitol of Bhubhaneswar. Khurda District has a population of approximately 1.9 million and is highly endemic for lymphatic filariasis caused by *Wuchereria bancrofti*, with surveys from 2001–2005 estimating between 22,500 and 235,000 microfilaria-infected persons [17]–[20]. CASA provides services to >20,000 lymphedema patients in the Orissa State. Study patients were enrolled from randomly selected villages in Bolagarh, one sub-district of Khurda district. The map shown in Figure 1 was generated by ArcMAP 10.1 software (ESRI, Redlands, California, USA), using shapefiles downloaded from DIVA-GIS (http://www.diva-gis.org/gdata).

### Study Design

The study was conducted from July 2009–July 2011 in 30 villages in Bolagarh sub-district. Villages were eligible for inclusion in the study if they had not yet been enrolled in the lymphedema management program, and were not located in the immediate vicinity of a village that had already been enrolled in the program. Lymphedema patients were selected based on a house-to-house morbidity census conducted by CASA in 2003 and repeated prior to the start of the program. Patients were eligible for the study if they were ≥14 years of age and had reported lower leg swelling of at least three months duration. Patients with lymphedema of the breast, arm, or genitals (in the absence of lower-limb lymphedema) were not eligible for participation in the study.

The study was powered to detect a 5% decrease in the frequency of ADL episodes, with a 15% drop-out rate, from baseline to 24 months post-enrollment in the lymphedema management program, with an alpha of 0.05.

In-person interviews with participating patients were conducted by trained local interviewers in Oriya, the local language. Interviews included questions regarding general demographic information, history of lymphedema, understanding of and compliance with lymphedema management, frequency of ADL episodes, and access to care.

Lymphedema patients were evaluated prior to enrollment in the lymphedema management program, and again at 1 month, 2 months, 3 months, 6 months, 12 months, 18 months, and 24 months after enrollment in the program. Evaluation included a physical examination of the affected extremity and administration of a pre-tested questionnaire. Due to logistics issues, the 18 month data collection was not performed on time and therefore is not included in this analysis.

The physical assessment of each patient was conducted by both a trained interviewer and a supervisor. Both the interviewer and the supervisor performed independent staging of the leg(s) and photographs were taken of the affected limb(s). Staff used the 7-stage classification system developed by Dreyer and colleagues [5] to stage patients' degree of lymphedema. Where staging was inconsistent between the interviewer and supervisor, or with prior or subsequent staging, photographs were independently reviewed by two physicians with extensive LF experience (P. Budge and L. Fox), and discrepancies were resolved. For this analysis, stages were combined into three categories: early lymphedema (stages 1–2), moderate lymphedema (stage 3), and advanced lymphedema (stages 4–7). An adenolymphangitis (ADL) episode was defined as a patient self-report of two of more of the following symptoms: redness, pain, or swelling of the leg or foot, with or without the presence of fever or chills, during any point in the previous 30 days.

### WHO Disability Assessment Schedule II

The World Health Organization Disability Assessment Schedule II (WHO DAS II) was administered to patients at each interview. The WHO-DAS II survey, created by the World Health Organization, is designed to assess daily function across six broad categories, or domains, including cognition, mobility, self-care, getting along with others, life activities, and participation in society [16]. The instrument measures an individual's perception of their disability through a series of questions scored on a 5-point scale ranging from 1 (“No difficulty”) to 5 (“Extreme difficulty or cannot do”). The questions are based on the interviewee's perception of their experiences over the last 30 days. Taken together, these scores provide an overall assessment of total perceived disability, with higher scores corresponding to higher levels of perceived disability. This analysis used simple (un-weighted) scoring of the WHO-DAS II domains to calculate an overall disability score.

### Data Analysis

Data were independently dual-entered into Epi Info 7, (Stone Mountain, 2008) and then checked for inconsistencies. Data cleaning and analysis were performed in SAS 9.3 (Cary, North Carolina, USA). Paired T-tests were used to examine perceived disability changes over time and changes in mean days of work lost due to lymphedema. These paired analyses compared the disability or domain score at each time point to the same patients' scores at baseline—the baseline scores of patients not present at any given assessment were not included in that assessment's comparison. Mixed effects model linear regression was used to identify factors associated with changes in disability scores over time, taking into account correlations in the data over the entire 24 month study period. All variables that were statistically significant (*P*≤0.05) on univariate analysis were included in the final predictive model, as were important demographic variables. Variables were checked for co-linearity before their inclusion in the final model.

### Sensitivity Analyses

To examine the effect of loss to follow-up, sensitivity analyses using the methods listed above, but excluding those patients not present at study end, were performed.

## Results

### Demographic Characteristics

A total of 457 patients were selected from 30 villages. Initially, 375 (82%) met the inclusion criteria and agreed to participate in the study. Five patients were subsequently excluded from analysis, due to lack of lymphedema on examination (n = 2), failure to meet the age criteria (n = 1), or mislabeling of survey forms (n = 2). Fifty-four (14.6%) patients were lost to follow-up during the 24 month study period. Over the course of the study, reasons for non-participation at any particular assessment were absence from the village at the time of the assessment (70%), refusal (7%), illness (6%), or death (17%). In total, the study encompassed over 658 person-years of observation time (baseline to time of last follow-up).

At enrollment participants averaged 57.2 years of age, and the majority were women (218, 59%) (**Table 1**). Most participants (298, 81%) were married, and only 75 (20%) had more than a primary school education. Approximately half of the study population (49%) identified “Homemaker/Housekeeper” as their primary occupation, while 57 (17%) reported being unemployed or retired. More than 40% (162) of patients reported at least one chronic health condition other than lymphedema at one or more time points during the study. The most commonly reported chronic conditions were gastrointestinal problems (18%) and high blood pressure (17%).

**Table 1:** Characteristics of patients enrolled in lymphedema management: July 2009–July 2011.

	Baseline (N = 370)		12 months (N = 320)		24 months (N = 315)		
Parameter	N	%	N	%	N	%	P value*
**Age** (Mean, SD)	57.15	13.94	57.07	13.62	56.96	13.58	0.6395
**Female Gender**	218	58.92	190	59.19	185	58.54	0.9209
**Married**	298	80.54	256	80	254	80.38	0.9578
**Higher than Primary Education**	75	20.33	61	19	63	19.94	0.8995
**Work**							
Homemaker/Housekeeper	165	49.25	148	50.17	147	52.69	0.5155
Unemployed/Retired	57	17.01	47	15.93	50	17.92	
Work or Study	113	33.73	100	33.9	82	29.39	
**Caste**							
General Caste	147	39.73	127	39.56	132	41.77	0.9033
Other Backward	175	47.3	153	47.66	147	46.42	
Scheduled Caste	28	7.57	25	7.79	20	6.33	
Scheduled Tribe	20	5.41	16	4.98	17	5.38	
**Any Chronic Health Condition**	162	43.78	136	42.37	131	41.46	0.733
**Co-morbidities**							
High Blood Pressure	62	16.76	65	20.25	62	19.62	0.3321
Diabetes	12	3.24	10	3.12	7	2.22	0.4165
Cancer	2	0.54	0	0	0	0	0.9761
Heart Problems	8	2.16	6	1.87	2	0.63	0.1178
Stomach Problems	66	17.84	62	19.31	71	22.47	0.1317
**Stage of Most-Effected Leg**							
Early (Stage 1–2)	184	49.73	177	55.31	188	59.49	**0.0155**
Moderate (Stage 3)	133	35.95	92	28.75	82	25.95	
Advanced (Stage 4–6)	53	14.32	51	15.94	46	14.56	
**Years with lymphedema**							
Mean, SD	25.48	16.04	25.77	16.37	25.23	16.05	0.9844
**Bilateral Lymphedema**	124	33.51	109	34.06	119	37.66	0.2585

* Denotes the p-value for the difference between baseline and 24 months.

The majority of patients had lymphedema classified as “early” (Stage 1–2) (50%), or “moderate” (Stage 3) (36%). Only 53 (14%) of patients had lymphedema classified as “advanced” (Stages 4–7). Patients reported having experienced lymphedema symptoms for an average of 25.5 years (range: 1.0–75.0 years). One hundred twenty-four (34%) patients reported bilateral lymphedema. There were no statistically significant differences in demographic characteristics between baseline and the twenty-four month assessment except that significantly more patients were classified as having early lymphedema at 24 months as compared to baseline (p = 0.0155). A subset analysis of the 316 patients present at 24 months revealed that 55 of these patients (17%) were in a lower stage category at study end compared to baseline, while 20 (6%) were in a higher stage category (data not shown). Among the 54 patients lost to follow-up by study end, 32 (59%) had early lymphedema, 15 (28%) had moderate lymphedema, and 7 (13%) had advanced lymphedema (data not shown). This did not vary significantly from the baseline characteristics of those who remained in the study.

### Perceived Disability Scores over Time

**Figure 2:**
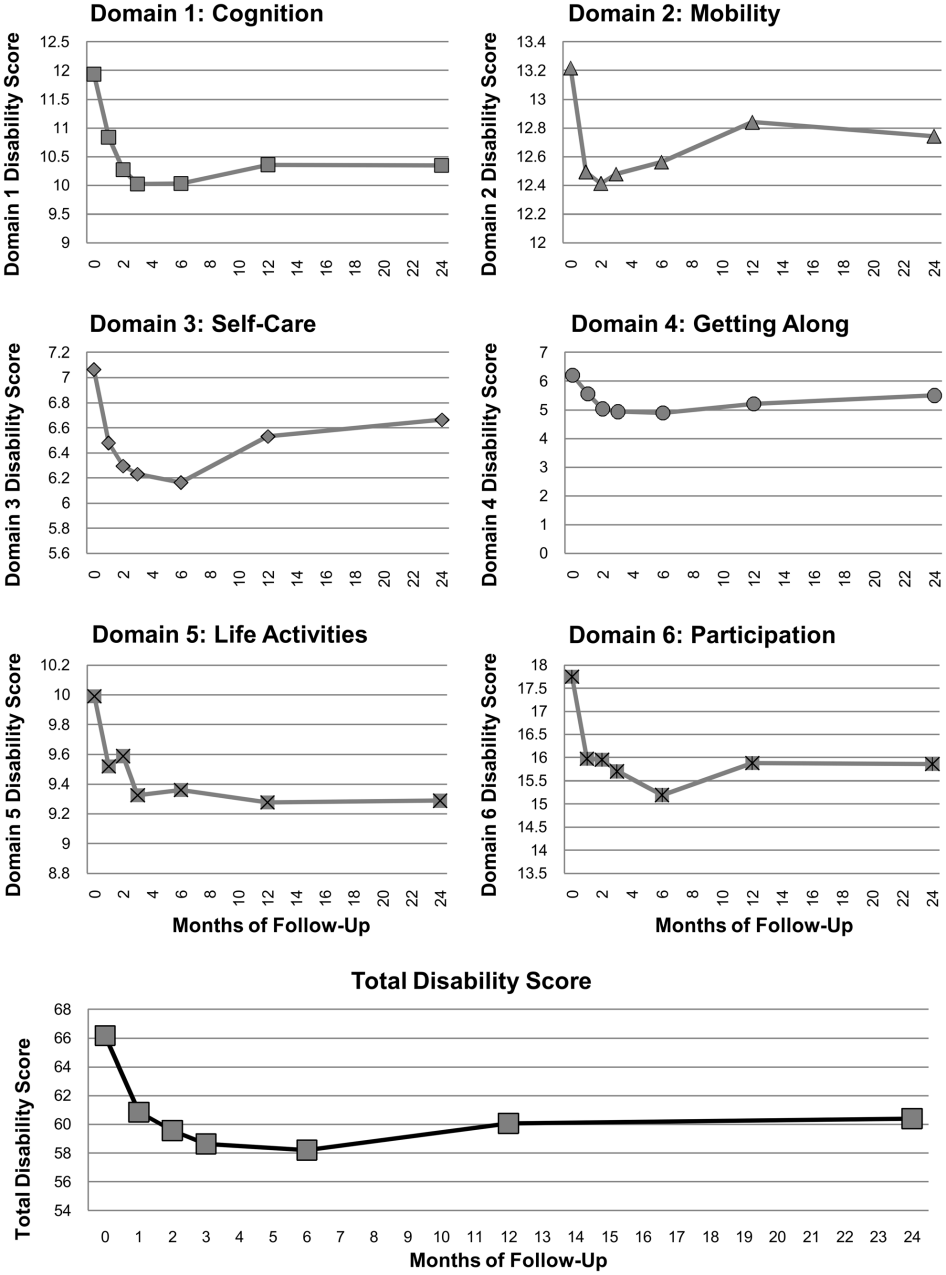
Mean WHO-DAS II disability scores by domain. Scores for each domain are shown as labeled; total disability is shown in the lowest panel.

Composite disability scores from the WHO-DAS II questionnaire decreased from an average score of 66.2 at baseline to 60.4 at 24 months (p<0.0001), a decline of more than 9%. This reflects significant and sustained reduction in each of the six WHO-DAS II component domains, with the exception of mobility and self-care (**Figure 2**). Patients reported a 13% decrease in cognitive disability from baseline to twenty-four months post-enrollment (p<0.0001). Disability in the domain “Getting Along with Others” declined 12% (P<0.005), while disability in life activities decreased 7% (p = 0.0046). Difficulty participating in society decreased 11% (p<0.0001) from baseline to twenty-four months. Disability in mobility also decreased slightly during the follow-up period (4%), as did scores for self-care (6%), though neither of these declines was statistically significant.

**Figure 3:**
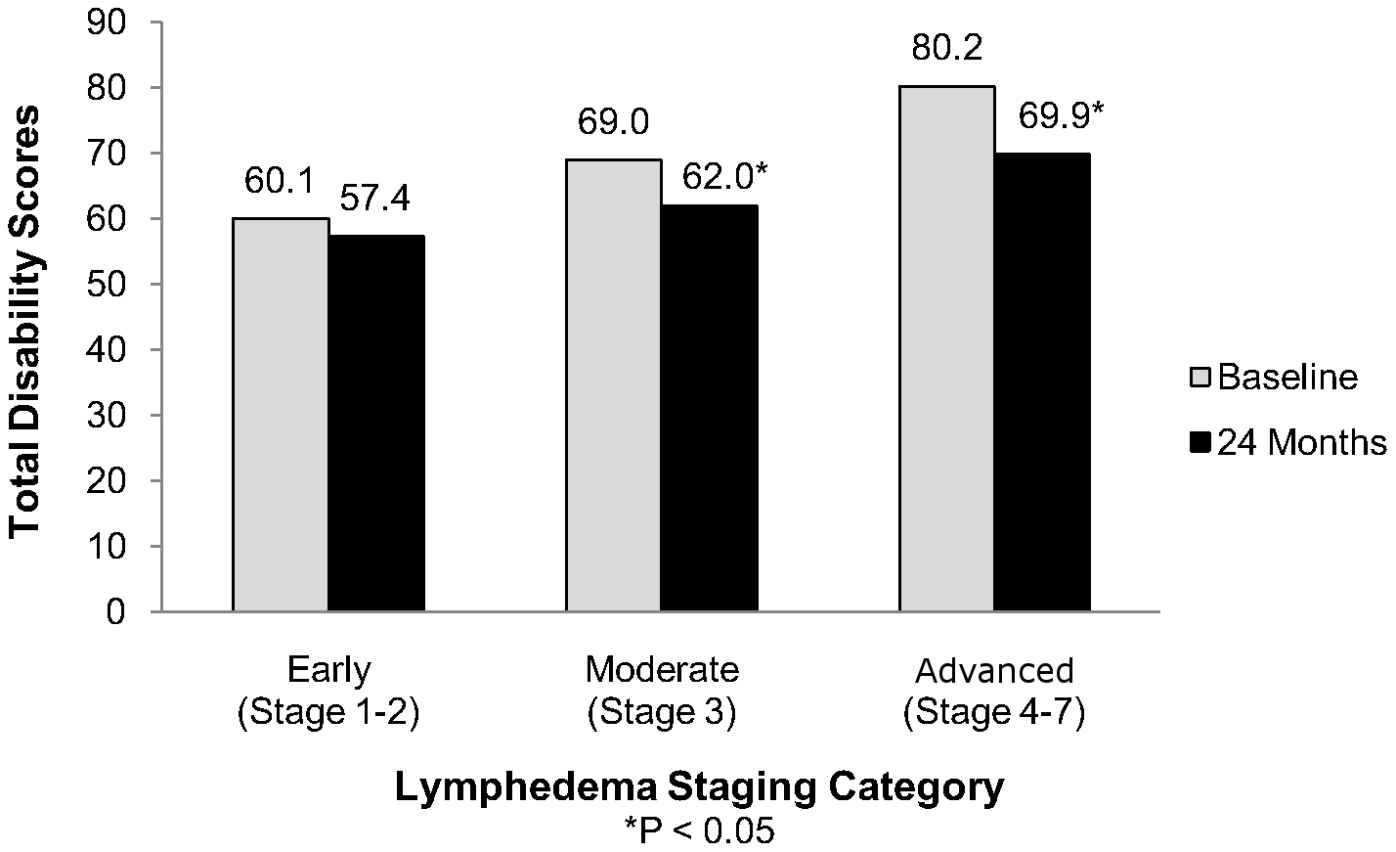
Total perceived disability scores. Total WHO DAS II scores (composite of all 6 domains) are shown prior to enrollment and 24 months after enrollment in the lymphedema management program, by lymphedema stage category.

After stratifying by lymphedema category, patients with the most advanced lymphedema (Stages 4–7) saw the largest reductions in overall disability scores (**Figure 3**). Scores in this group fell approximately 13% between baseline and 24 months (p = 0.0044). Patients with moderate lymphedema (Stage 3) reported a 10% drop in disability over 24 months (p = 0.0011), while patients with early stage lymphedema (Stages 1–2) experienced a smaller percent reduction in scores (5%) that did not reach statistical significance (p = 0.0697).

### Analysis of Factors Affecting Perceived Disability

A number of factors were significantly associated with total disability score on univariate analysis (**Table 2**). Factors associated with a decrease in perceived disability and lower WHO-DAS II composite scores included having at least a primary school education (estimate: −6.9, 95% CI: −10.4, −3.4), and being employed as a homemaker (estimate: −5.6, 95% CI: −7.9, −3.4) or a worker or student (estimate: −7.6, 95% CI: −10.0, −5.3). Being currently married was also associated with a lower disability score (estimate: −3.1, 95% CI: −5.7, −0.6). The only individual component of lymphedema management that was significantly associated with reduced disability levels on univariate analysis was wearing shoes. Compared to those never wearing shoes, individuals reporting always wearing shoes while outside had disability scores 2.8 points lower (95% CI: −4.6, −1.1). When compared to baseline measures, time in the program was also associated with decreased disability scores for every time-point.

**Table 2:** Univariate (unadjusted) and multivariate (adjusted) analysis factors associated with WHO-DAS II disability scores.

	Unadjusted		Adjusted	
Variable	Estimate	95% CI	Estimate	95% CI
**Age Quartile**				
<45 Years	ref	ref	ref	ref
45–57	2.62	(−1.29, 6.54)	1.37	(−1.89, 4.62)
>57–69	2.92	(−0.98, 6.83)	2.20	(−1.35, 5.74)
>70	**10.89**	**(7.03, 14.76)**	**7.89**	**(4.04, 11.74)**
**Female Gender**	2.85	(−0.07, 5.76)	**5.73**	**(2.18, 9.27)**
**Higher than Primary Education**	**−6.88**	**(−10.40, −3.35)**	−1.25	(−4.47, 1.98)
**Work**				
Unemployed or Retired	ref	ref	ref	ref
Homemaker or Housekeeper	**−5.63**	**(−7.86, −3.40)**	**−5.76**	**(−8.22, −3.29)**
Work or Study	**−7.63**	**(−9.97, −5.30)**	**−4.46**	**(−6.84, −2.08)**
**Married**	**−3.14**	**(−5.69, −0.58)**	−1.29	(−3.84, 1.25)
**Caste**				
General Caste	ref	ref	ref	ref
Other Backward	0.76	(−2.34, 3.86)	1.43	(−1.10, 3.97)
Scheduled Caste	−2.61	(−8.35, 3.12)	1.85	(−2.80, 6.50)
Scheduled Tribe	2.12	(−4.57, 8.82)	4.69	(−0.99, 10.37)
**Any Chronic Health Condition**	**7.18**	**(4.27, 10.08)**	**5.45**	**(2.98, 7.92)**
**Stage of Most-Affected Leg**				
Early (Stage 1–2)	ref	ref	ref	ref
Moderate (Stage 3)	**2.74**	**(0.91, 4.57)**	**1.91**	**(0.12, 3.71)**
Advanced (Stage 4–6)	**11.66**	**(8.34, 14.97)**	**8.38**	**(4.97, 11.80)**
**Bilateral Lymphedema**	**3.50**	**(0.68, 6.32)**	−0.40	(−2.84, 2.04)
**Washing with Soap**				
Never	ref	ref	ref	ref
Less than Daily	−3.01	(−6.68, 0.65)	−3.63	(−7.55, 0.29)
At least Daily	−2.76	(−6.52, 1.00)	−2.21	(−6.24, 1.82)
**Exercise**				
Never	ref	ref	ref	ref
Less than Daily	−2.38	(−4.89, 0.14)	**−2.64**	**(−5.17, −0.10)**
At least Daily	−0.47	(−1.99, 1.06)	0.44	(−1.11, 1.99)
**Elevation**				
Never	ref	ref	ref	ref
Less than Daily	−1.35	(−6.09, 3.39)	−0.43	(−4.98, 4.12)
At least Daily	0.17	(−1.67, 2.01)	0.34	(−1.53, 2.21)
**Wear Shoes Outside**				
Never	ref	ref	ref	ref
Often	−0.75	(−2.60, 1.11)	0.44	(−1.37, 2.25)
All of the Time	**−2.80**	**(−4.56, −1.05)**	−0.10	(−1.81, 1.61)
**Cream Use**				
Never	ref	ref	ref	ref
Often	−0.69	(−3.23, 1.84)	−0.05	(−2.50, 2.40)
All of the Time	1.23	(−0.21, 2.66)	0.80	(−0.64, 2.25)
**Any Interdigital Lesions**	**5.06**	**(3.30, 6.82)**	**2.68**	**(0.85, 4.52)**
**ADL Episode in the Past 30 Days**	**11.49**	**(9.98, 13.00)**	**10.63**	**(9.10, 12.16)**
**Time**				
Baseline	ref	ref	ref	ref
1 Month	**−5.06**	**(−7.33, −2.79)**	**−4.64**	**(−8.00, −1.29)**
2 Months	**−6.44**	**(−8.74, −4.14)**	**−5.72**	**(−9.25, −2.20)**
3 Months	**−7.26**	**(−9.52, −5.00)**	**−6.21**	**(−9.74, −2.68)**
6 Months	**−8.41**	**(−10.56, −6.25)**	**−6.33**	**(−9.87, −2.80)**
12 Months	**−6.18**	**(−8.43, −3.92)**	**−4.69**	**(−8.21, −1.17)**
24 Months	**−5.68**	**(−8.39, −2.98)**	−3.67	(−7.48, 0.14)
**Perceived Health Status in the Past 30 Days**			–	–
Very good	ref	ref	–	–
Good	**7.96**	**(4.83, 11.10)**	–	–
Moderate	**15.77**	**(12.55, 18.99)**	–	–
Bad	**28.68**	**(25.26, 32.10)**	–	–
Very bad	**38.85**	**(33.10, 44.61)**	–	–

Factors associated with an increased disability score in univariate analysis included belonging to the highest age quartile (estimate: 10.9, 95% CI: 7.0, 14.8), the presence of one or more additional chronic health problems (estimate: 7.2; 95% CI: 4.3, 10.1), moderate (estimate: 2.7; 95% CI: 0.9, 4.6) or advanced lymphedema (estimate: 11.7, 95% CI: 8.3, 15.0), bilateral lymphedema (estimate: 3.5, 95% CI: 0.7, 6.3), the presence of interdigital lesions, which are fungal and bacterial infections in the interdigital web spaces, (estimate: 5.1, 95% CI: 3.3, 6.8), and having had an ADL episode in the previous 30 days (estimate: 11.5, 95% CI: 10.0, 13.0). The strongest predictor of perceived disability, however, was patients' self-reported health rating for the past 30 days. Patients reporting “Very bad” health had scores more than 38 points higher than those reporting “Very good” health (estimate: 38.9, 95% CI: 33.1, 44.6).

In multivariate analysis several factors remained significantly associated with decreased disability scores on the WHO-DAS II (**Table 2**). After controlling for covariates, patients who reported being employed as a homemaker had WHO-DAS II scores 5.7 points lower than those who were unemployed (95% CI: −8.2, −3.3), while worker/student had scores that were 4.5 points lower than those who were unemployed (95% CI: −6.8, −2.1). The individual components of lymphedema management, including soap use, elevation of the affected limb, wearing shoes, and antifungal cream use were not significantly associated with disability scores after controlling for other covariates. However, patients who reported performing leg exercises more than once a week (but less than once a day) had scores 2.6 points lower than patients who never performed the exercises (95% CI: −5.2, −0.1). Time enrolled in the program was significantly associated with decreased disability scores through 12 months of program participation.

Risk factors for increased overall disability that remained significant in multivariate analysis included belonging to the oldest age quartile, female gender, the presence of other chronic health problems, moderate or advanced lymphedema, the presence of interdigital lesions, and having had an ADL episode in the past 30 days. Patients belonging to the oldest age quartile had scores 7.9 points higher than patients in the youngest age quartile (95% CI: 4.0, 11.7), while women scored 5.7 points higher than their male counterparts (95% CI: 2.2, 9.3). Patients with lymphedema stage 4 or higher scored 8.4 points higher on the WHO-DAS II than patients with early stage lymphedema (95% CI: 5.0, 11.8). ADL episodes had the largest effect on disability scores in our model. Patients reporting an ADL episode in the previous 30 days had scores 10.6 points higher than those who had not reported an ADL episode (95% CI: 9.1, 12.2).

In a multivariate model including the predictors above as well as patient self-reported overall health status, patient health rating during the previous 30 days remained the largest predictor of increased disability (data not shown). After controlling for covariates, patients reporting “Very bad” health during the last 30 days scored approximately 33 points higher on total disability than patients who reported “Very good” health (95% CI: 27.2, 39.4).

### Days of Work Lost Due to Lymphedema

**Figure 4:**
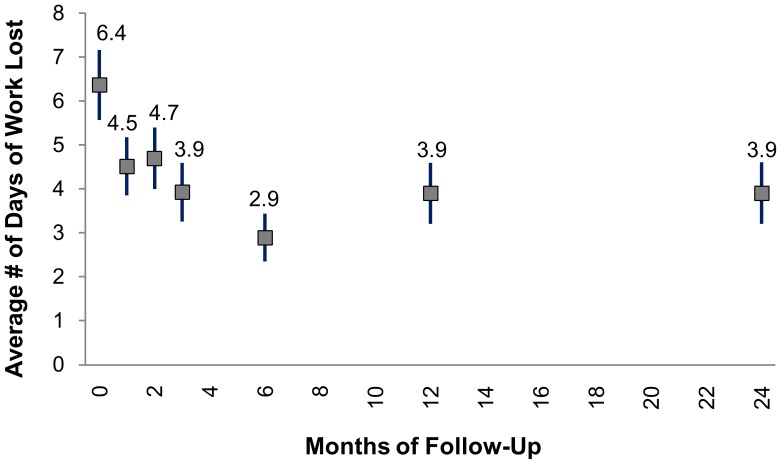
Reported days work lost. Self-report of the mean number of days of work lost due to lymphedema-related disability in the past 30 days. Error bars represent 95% confidence intervals around the mean. All times were significantly different from baseline (time 0).

At each assessment time point, patients were asked about the number of days of work lost in the preceding 30 days due to lymphedema-associated disability. At baseline, patients reported an average of 6.4 (95% CI: 5.6, 7.2) days of work lost due to disability in the previous 30 days (**Figure 4**). After enrollment into the lymphedema management program, the average number of days of work lost in the previous 30 days declined to 4.7 (95% CI: 4.0, 5.4) at 2 months and 2.9 (95% CI: 2.4, 3.4) at 6 months. At 24 months post-enrollment, the number of days of work lost remained significantly lower than baseline, at 3.9 (95% CI: 3.2, 4.6).

Stratified by lymphedema stage, patients with advanced lymphedema reported missing more days of work due to their lymphedema in the previous 30 days at baseline than patients with early stage lymphedema (5.3 days vs. 10.4 days, p = 0.0265) (Data not shown). However, patients with advanced lymphedema also saw the greatest reduction in days of work lost at 24 months, with a 44% decline from 10.4 to 5.9 days (p = 0.0083). Patients with moderate stage lymphedema also saw a significant decrease in days of work lost from 6.2 to 4.5 days. (28%, p = 0.0439).

### Sensitivity Analysis

The mixed effects model used to analyze factors associated with a change in disability score and the paired comparisons of perceived disability (comparing each individual's score to their corresponding baseline score) account for missing data, so exclusion of the 54 patients not present at study end should have little effect on the reported outcomes. To verify this, the analyses were repeated including only those 316 patients present at study end. This did not change the significance of any observed differences in perceived disability, and in all cases exaggerated the magnitude of the difference (data not shown). Excluding the 54 patients not present at study end also made no difference in determining which variables were significant in the multivariate analysis, except to make the association between the 24 month assessment (variable “Time” in Table 1) reach statistical significance (data not shown).

## Discussion

This study found that patients enrolled in a community-based lymphedema management program experienced less disability in almost every domain of the WHO-DAS II, including participation in community life and cognition, compared to baseline. These benefits were sustained through two years of follow-up. These results are consistent with other studies which have demonstrated decreased disability, fewer ADL episodes, and improved quality of life amongst lymphedema patients involved in lymphedema management programs [21]–[23].

After controlling for other predictors, including time in the program, the best predictor of patient perceived disability was self-reported health status in the previous 30 days. Worsening reported health status corresponded with increases in WHO-DAS II composite disability scores at every time point in the survey, indicating that self-reported health status may serve as a simple proxy measure for composite perceived disability among lymphedema patients.

Other significant contributors to increases in composite disability scores were older age and advanced lymphedema. Though this study found that the oldest patients had the highest disability scores at both baseline and 24 months after enrollment, these patients also experienced the greatest percent declines in these scores over their two years in the program. This same pattern held true for patients with the most advanced lymphedema (Stages 4–7), who experienced greater percent declines in their composite disability scores than patients with less advanced lymphedema (Stage 1–2). These findings suggest that simple, low-cost interventions, such as those used in this community-based lymphedema management program, can significantly impact perceived levels of disability for even the oldest and most advanced lymphedema patients, even in the absence of more intensive and costly interventions such as bandaging, massage, and IV antibiotics.

Younger patients and those with early stage lymphedema and lower levels of disability at enrollment also perceived less disability after participation in the lymphedema management program. The findings suggest that early exposure to lymphedema management may substantially decrease perceived disability among patients in the early stages of lymphedema. As community-based lymphedema management programs expand, an emphasis on prompt enrollment of patients with stage 1 or 2 lymphedema will be particularly important. Increased community awareness of LF and efforts to reduce stigma will likely be vital to the identification and enrollment of early stage lymphedema patients.

After controlling for other predictors, reporting an ADL episode in the previous 30 days remained a significant predictor of increased disability. Prior research has demonstrated that the prevention of ADL episodes slows long-term lymphedema progression [24], [25]; our data demonstrate that reducing the number of ADL episodes also decreases patients' perceived level of disability. Our results suggest that lymphedema management techniques that assist in preventing ADL episodes can reduce patient disability as well as increase productivity and participation in society. While this study focuses on perceived disability as a surrogate for quality of life, it is interesting to note that the maximal reduction in perceived disability occurred between 2 and 6 months, a period reported by others as the time of maximum impact on ADLA episodes [26]. A more detailed analysis of the effect of this lymphedema management program on ADLA episodes is underway.

These reductions in disability can have significant economic impact for both the patients and their communities. At twenty-four months post-enrollment, patients in our study gained an average of 2.4 work days per month as the result of decreased perceived disability. If extrapolated to all 17,036 (Little KM, unpublished data) lymphedema patients in Khurda District, this translates into approximately 2,688 person-years of productive time gained over the course of the twenty-four month study period. These findings suggest that substantial economic gains for individuals, families, and communities would result from an increased emphasis on LF morbidity reduction, especially in high-prevalence areas. In India, where nearly 20 million individuals suffer from symptomatic LF infections [9], the economic benefits of lymphedema management programs in terms of work days saved would be considerable.

Although lymphedema management improved disability scores over time, advanced lymphedema, the presence of other chronic health conditions, and female gender remained strong predictors of worse perceived disability. Though patients with advanced lymphedema experienced the largest reduction in their perceived disability during the study, their composite disability scores remained higher than young or early stage patients for the entire follow-up period. While community-based lymphedema management programs may provide increased access to hygiene supplies and education, they are typically unable to offer patients with advanced lymphedema more expensive and intensive therapies such as bandaging, antibiotics, and a specialized referral infrastructure. Effective control of LF-related morbidity may require dual development of low-cost community interventions alongside more complex services for patients with advanced lymphedema and other chronic health conditions. Further research should explore the feasibility and effectiveness of these interventions in low-resource settings. Opportunities for collaboration with other chronic health programs including those focusing on diabetes, leprosy, and venous insufficiency, should also be explored. Finally, future work should focus on women's health in relation to lymphedema in an effort to reduce the disability gender-gap observed in this study.

### Limitations

This study had several limitations. First, survey results were based on patient recall and perceived disability during the previous 30 days and may be subject to recall bias. Patients enrolled in this study were included based on the presence of lymphedema in one or both legs. Because blood was not drawn to test for the presence of microfilaremia or filarial antigenemia the lymphedema management program may have enrolled patients with non-filarial lymphedema. Nevertheless, it is important to note that lymphedema management programs are recommended for lymphedema resulting from all causes. Additionally, the study is limited by the lack of a comparable control group not receiving the community-based lymphedema management program, as it is considered inappropriate to withhold knowledge of lymphedema management techniques from patients with lymphedema.

In order to account for repeat measurements over time, we used a mixed effects model that incorporated time as a variable in both the univariate and multivariate analyses. Because compliance with foot care was dramatically increased at all assessments subsequent to baseline, our model likely underestimates the effect of compliance with foot care on overall disability score. Indeed, compliance with foot care becomes highly significant when time is taken out of the model (data not shown). Finally, there was an increase in most WHO DAS II domain scores at 12 months compared to 6 months. It is not unexpected to see fluctuations in perceived disability from chronic diseases; more frequent or longer monitoring would provide a better sense of whether the benefits we have observed will be sustained. Future research will address the relationship between ADL episodes and lymphedema progression in this cohort.

### Conclusion

While the effects of lymphedema management on clinical disease, disability, and quality of life have been studied previously [6], [7], [14], [22], this is one of the first evaluations of a community-based lymphedema management program with 24 month longitudinal follow-up. Our findings indicate that community-based lymphedema management programs can reduce patient perceived disability and reduce the number of work days lost due to lymphedema symptoms. Significantly, these effects were maintained for two years following program enrollment. These data emphasize the need for national lymphatic filariasis elimination programs to prioritize morbidity management and disability prevention programs to improve the lives of those suffering from lymphedema associated with lymphatic filariasis.

## Supporting Information

Checklist S1 STROBE Checklist.
(DOC)
